# *‘I knew I'd be taken care of’*: Exploring patient experiences in the Emergency Department

**DOI:** 10.1111/jan.15317

**Published:** 2022-06-19

**Authors:** Claudia Bull, Sharon Latimer, Julia Crilly, David Spain, Brigid M. Gillespie

**Affiliations:** ^1^ School of Nursing and Midwifery Griffith University – Gold Coast campus Southport Queensland Australia; ^2^ School of Nursing and Midwifery Griffith University – Logan campus Logan Queensland Australia; ^3^ Gold Coast Hospital and Health Service Southport Queensland Australia; ^4^ NHMRC Centre of Research Excellence in Wiser Wounds, Menzies Health Institute Queensland Griffith University Southport Queensland Australia

**Keywords:** communication, emergency department, interviews, nursing, patient experience, qualitative research, relationships, shared decision‐making, waiting

## Abstract

**Aims:**

To explore adult Emergency Department patient experiences to inform the development of a new Emergency Department patient‐reported experience measure.

**Design:**

Descriptive, exploratory qualitative study using semi‐structured individual interviews with adult Emergency Department patients.

**Methodology:**

Participants were recruited across two Emergency Departments in Southeast Queensland, Australia during September and October 2020. Purposive sampling based on maximum variation was used. Participants were recruited during their Emergency Department presentation and interviewed in 2‐weeks via telephone. Inductive thematic analysis followed the approach proposed by Braun and Clarke (2012).

**Results:**

Thirty participants were interviewed, and four themes were inductively identified: *Caring relationships between patients and Emergency Department care providers*; *Being in the Emergency Department environment*; *Variations in waiting for care*; and *Having a companion in the Emergency Department*. *Caring relationships between patients and Emergency Department care providers* included being treated like a person and being cared for, being informed about and included in care, and feeling confident in care providers. *Being in the Emergency Department environment* included being around other patients, feeling comfortable and having privacy. *Variations in waiting for care* included expecting a longer wait, waiting throughout the Emergency Department journey and receiving timely care. *Having a companion in the Emergency Department* included not feeling alone, and observing care providers engage with companions.

**Conclusion:**

Patient experiences in the Emergency Department are multifaceted, and themes are not mutually exclusive. These findings demonstrate consistency with the core experiential themes identified in the international literature.

**Impact:**

Strategies to improve patient engagement in shared decision‐making, and communication between patients and care providers about wait times will be critical to optimizing Emergency Department patient experiences, and person‐centred practice. These findings holistically conceptualize patient experiences in the Emergency Department which is the first step to developing a new Emergency Department patient‐reported experience measure.

## 1 INTRODUCTION

Patient experiences––a description of what happened during a care encounter, and how it happened from the patients' perspective (Bull et al., 2019)––are unique and complex in the Emergency Department. A recent review identified that Emergency Department patient experiences can be broadly described by two interrelated themes: *Relationships between Emergency Department patients and care providers*, and *Spending time in the Emergency Department environment* (Bull et al., 2021). Other reviews have similarly demonstrated growing interest in this area by describing the determinants of patient experiences in the Emergency Department including, staff‐patient interactions (particularly related to communication and empathic care), wait times, the Emergency Department environment and the emotional impact of experiencing an emergency (Gordon et al., 2010; Graham et al., 2019; Sonis et al., 2018). Additionally, the psychometric properties of Emergency Department patient‐reported experience measures have been examined, highlighting few instruments with variable levels of validity and reliability (Male et al., 2017). However, despite international efforts to better understand and improve Emergency Department patient experiences, there has been minimal exploration of adult patient experiences in Australian Emergency Departments.

## 2 BACKGROUND

The rate of patient presentations to Australian Emergency Departments continues to exceed the rate of population growth. Burkett et al. identified that between 2006–07 and 2010–11, Emergency Department presentations increased by 12.63%, despite Australia's population increasing by only 7.26% (Burkett et al., 2017). The authors also predict that the rates of Emergency Department presentation will increase by 177% in the year 2050, with significantly higher numbers of presentations by individuals between the ages of 65–84 and ≥85 (242% and 411%, respectively) (Burkett et al., 2017). Given the reliance of many Australians on Emergency Department services, it is critical that there are robust mechanisms in place to measure and monitor the quality of patient experiences in the Emergency Department.

The Australian Commission on Safety and Quality in Health Care recognizes *Partnering with Consumers* as one of the eight key national safety and quality standards for health services (Australian Commission on Safety and Quality in Health Care, 2017). They note that partnering with consumers is critical to the planning, design, delivery, measurement and evaluation of systems and services (Australian Commission on Safety and Quality in Health Care, 2017). In their key strategies to promote the standard of *Partnering with Consumers*, the Australian Commission on Safety and Quality in Health Care prioritizes providing consumers with the opportunity to feedback on the safety and quality of services, specifically identifying surveys as a mechanism to achieve this. However, there is currently no nationally endorsed Emergency Department patient‐reported experience measure. Some Australian states have developed and implemented their own measures to monitor and evaluate Emergency Department patient experiences (Bureau of Health Information, 2021; Queensland Health, 2021; Victorian Agency for Health Information, 2021), but differences in the rigour of survey development, data collection practices and jurisdictional priorities for patient experiences impede national comparisons and benchmarking opportunities.

A critical process in survey development is determining what concepts to measure (DeVellis, 2017). In the context of patient‐reported experience measures, important concepts to measure are most meaningfully determined by the individuals whose experiences we are aiming to capture; that of patients. Not only does their involvement enhance the content validity of the instrument (i.e. the degree to which the instrument adequately reflects the construct(s) being measured) and grounding of items in real life (Boateng et al., 2018; Mokkink et al., 2018), but it ensures that subsequent service improvement efforts are person‐centred, as they are guided by patient preferences and values for optimal care experiences.

## 3 THE STUDY

### 3.1 Aim

The purpose of this study was to explore adult Emergency Department patient experiences to inform the development of a new Emergency Department patient‐reported experience measure.

### 3.2 Design

This descriptive, exploratory qualitative study gathered data from adult Emergency Department patients using individual, semi‐structured telephone interviews. The study was underpinned by a qualitative constructivist‐interpretivist paradigm (Ponterotto, 2005). The constructivist‐interpretivist paradigm was chosen because it is based on a relativist ontological position and a subjective epistemological position (Scotland, 2012). A relativist ontological position is the belief that reality is subjective and differs from one person to the next. A subjective epistemological position is the belief that people construct meaning in different ways because we each interact with the world individually. Thus, the constructivist‐interpretivist paradigm was suitable to underpin this study given its exploratory nature.

### 3.3 Sample/participants

The study was conducted during September and October 2020 at two public hospital Emergency Departments in Southeast Queensland, Australia. Potential study participants were recruited using a predetermined purposive sampling frame based on maximum variation for age, gender, reason for presentation and the Emergency Department presented to (Palinkas et al., 2015). Maximum variation sampling was used to capture a range of participant perspectives, and provide a holistic understanding of the phenomenon being explored by diversifying the recruited participants on select characteristics. As the findings of this study will inform the development of an Emergency Department patient‐reported experience measure, a broad spectrum of experiences is desirable to ensure that the instrument is generalizable to all adult Emergency Department populations. Participant eligibility criteria is described in Table 1.

**TABLE 1:** Study participant eligibility

Eligible participants:	Ineligible participants:
Aged ≥18 years old In the Emergency Department between 21 September and 4 October 2020 Able to speak, read and comprehend English Able to provide written consent at the time of recruitment, and Willing to undertake a telephone interview in 2 weeks of their Emergency Department presentation	Required a translator Unconscious or semi‐conscious for most of their Emergency Department presentation Triaged as a category 1 patient using the Australasian Triage Scale (i.e. immediately life‐threatening)^a^ Transported to the Emergency Department by police or correctional services Presented to the Emergency Department for mental health reasons Unsafe to approach at recruitment (including patients suspected of having COVID‐19), or Unable to undertake a telephone interview in 2 weeks of their Emergency Department presentation

^a^ Australasian College for Emergency Medicine. Triage. ACEM. https://acem.org.au/Content‐Sources/Advancing‐Emergency‐Medicine/Better‐Outcomes‐for‐Patients/Triage. Published 2020. Accessed 18 Aug, 2020.

**FIGURE 1:**
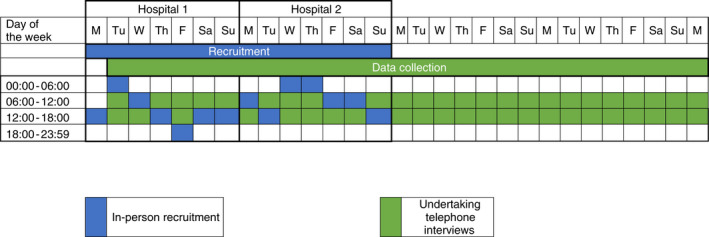
Study recruitment and data collection schedule. Legend: M = Monday; Tu = Tuesday; W = Wednesday; Th = Thursday; F = Friday; Sa = Saturday; Su = Sunday

Potential participants were recruited face‐to‐face during randomly allocated 6‐h recruitment shifts, across seven consecutive days per site (Figure 1). During recruitment shifts, eligible patients were approached by an Emergency Department physician or nurse after treatment had commenced (but prior to discharge or transfer), and asked to provide written consent if they agreed to be approached by the researcher (CB). Consenting patients were then approached, informed of the study and invited to participate in a telephone interview in the next 2 weeks. Consenting participants provided the recruiting researcher with their first and last name, best contact number and identified suitable days and times to be interviewed. The recruiting researcher made field notes during recruitment shifts about the Emergency Department environment, observations about patients and staff, and biases and assumptions she reflected on throughout recruitment.

### 3.4 Data collection

An interview guide (Appendix 1) was developed by the research team to assist the flow of conversation, which was pilot tested with four individuals who had recently presented to the Emergency Department (interviews were not included in analysis). Interview guide questions were informed by a systematic mixed studies review of the international literature describing patient experiences in the Emergency Department (Bull et al., 2021). One researcher (CB) undertook all interviews, which were audio recorded. Regular meetings were held with the research team to discuss emerging findings. All members of the team also had access the interviewing researchers' contact summaries from each interview. Contact summaries detailed the main themes discussed in the interview, whether the interview raised new thoughts or questions for upcoming interviews, and additional comments that the interviewing researcher noted (e.g. participants tone of voice and level of detail provided). As such, the research team collectively agreed when data saturation was evident (i.e. when no new ideas emerged throughout interviews). No repeat interviews were undertaken, and transcripts were not returned to participants for member checking.

With participant consent, the following demographic information was extracted from participants' Emergency Department electronic health records: age, gender, diagnosis (as described by the Emergency Department ICD‐10‐AM Principal Diagnosis Short List [IHPA, 2019]), Australasian Triage Scale categorisation, mode of arrival to the Emergency Department, duration of wait between arrival to the Emergency Department and treatment commencement, duration of the entire Emergency Department visit and Emergency Department end status.

### 3.5 Ethical considerations

Ethics clearance was given by the relevant institutions (Ref No: HREC/2020/QGC/61674 and 2020/444). All participants provided written consent to participate in the study.

### 3.6 Data analysis

Preliminary data analysis occurred simultaneous to data collection, enabling constant comparison and the identification of data saturation (Whitehead et al., 2013). During interviews, notes were made about participant Emergency Department experiences. In between interviews, contact summaries were completed to guide subsequent interviews and to prompt for emerging ideas. All interviews were transcribed verbatim, cleaned and removed of extraneous content.

An inductive thematic analysis approach was used to formally analyse interview transcripts. Analysis occurred in an iterative manner to develop subthemes and themes, following the six‐step approach proposed by Braun and Clarke (Braun & Clarke, 2006, 2012). This included: (i) data familiarization; (ii) line‐by‐line coding; (iii) collating codes into potential themes; (iv) generating thematic maps; (v) refining and defining themes and sub‐themes; and (vi) extracting descriptions from interviews and generating results (Braun & Clarke, 2006). Regular meetings were held by the research team to achieve a jointly developed interpretation of the data.

### 3.7 Rigour

The research team members have backgrounds and expertise in nursing, nutrition, emergency care, research and patient safety. The first author (CB) undertook all interviews. She has an honours degree and this study forms part of her PhD candidature. All authors are experienced in qualitative research.

This study was guided by Koch's (2006) criteria for establishing trustworthiness in qualitative inquiry (Koch, 2006). Trustworthiness was established by demonstrating reflexivity, credibility, transferability and dependability (Koch, 2006). Table 2 describes how these criteria were achieved.

**TABLE 2:** Demonstrating trustworthiness in qualitative data collection and analysis

Trustworthiness criteria	Fulfilment of criteria
Reflexivity	The first author maintained a field journal noting her interactions with staff and patients in the Emergency Department, observations about the Emergency Department environment, and important considerations, biases and assumptions that arose during participant recruitment, data collection and analysis (which were discussed in team meetings).
Credibility	All members of the research team were involved in the analysis process to establish consistency in the interpretation of the data.^a^
Transferability	By using a maximum variation sampling frame and recruiting participants at random times in the Emergency Department, the experiences from a broad spectrum of patients were captured.^b^
Dependability	The use of contact summaries, maintaining a strong audit trail and holding regular team meetings throughout the data collection and analysis periods

^a^ Miles MB, Huberman AM. *Qualitative data analysis: An expanded source book*. 2nd ed. California: Thousand Oaks; 1994.

^b^ Sandelowski M. The problem of rigor in qualitative research. *ANS Adv Nurs Sci*. 1986;8(3):27‐37.

Findings are reported using themes and sub‐themes. Direct extracts from interview transcripts are included to support the findings and strengthen dependability. Participant labels are based on participant number: gender: age to maintain participant anonymity.

## 4 FINDINGS

### 4.1 Participants

Thirty participants were interviewed; their characteristics are presented in Table 3. The sample included equal numbers of males and females, whose median age was 54.5 years. Approximately 50% of the sample presented to the Emergency Department with an injury (e.g. fractures, open wounds, muscle/tendon damage, superficial injuries or musculoskeletal injuries). One quarter of participants presented with symptoms related to the cardiovascular system. The median time participants waited to receive meaningful treatment was 39.5 min (IQR: 44 min), and the median Emergency Department length of stay was under 6 h. Close to 75% of the sample was discharged to their usual place of residence following their Emergency Department episode. Interviews lasted between 17 and 55 min (average time: 34 min).

**TABLE 3:** Participant demographic and presentation‐related characteristics

	Median (interquartile range)
Age (years)	54.5 (25.5)
Time to meaningful treatment	39.5 mins (44 mins)
Emergency Department length of stay	5 h, 48 min (3 h, 2 min)
	**n (%)**
Hospital	
#1	20 (66.6%)
#2	10 (33.3%)
Gender	
Female	15 (50.0%)
Male	15 (50.0%)
Principal diagnosisa^a^	
*Injury* (including fracture, open wound, muscle/tendon, superficial and musculoskeletal)	15 (50.0%)
*Cardiovascular* (e.g. chest pain, hypertension, Atrial Fibrillation)	8 (26.7%)
*Digestive* (e.g. biliary colic, abdominal pain, Gastrointestinal bleed)	3 (10.0%)
*Genitourinary* (e.g. urinary tract infection, Bartholin's abscess, renal stones)	3 (10.0%)
*General symptoms* (e.g. syncope, presyncope)	1 (3.3%)
Triage category	
2	11 (36.7%)
3	8 (26.6%)
4	11 (36.7%)
5	0 (0%)
Mode of arrival to Emergency Department	
Self‐presented	21 (70%)
Brought in by ambulance	9 (30%)
Emergency Department end status	
Discharged	22 (73.3%)
Admitted	8 (26.7%)

Abbreviation: IQR, Interquartile range.

^a^ Totals >30 diagnoses as some participants had >1 diagnoses.

### 4.2 Qualitative interview findings

Four themes were inductively identified from the qualitative data: (i) Caring relationships between patients and Emergency Department care providers; (ii) Being in the Emergency Department environment; (iii) Variations in waiting for care; and (iv) Having a companion in the Emergency Department. Themes and sub‐themes (Table 4) overlapped in how they contributed to patient experiences in the Emergency Department.

**TABLE 4:** Themes and sub‐themes of patient experiences in the emergency department, including exemplar quotes from participant interviews

Themes	Sub‐themes	Exemplar quotes from participant interviews
Caring relationships between patients and Emergency Department care providers	Being treated like a person and being cared for	**Quote 1:** *‘He straight away put me at ease and made me feel okay about that [taking up smoking again despite a previous heart attack], and said that nobody's perfect, we are all human, everybody makes mistakes, and how good it was that I had been able to give up again. I really appreciated that sort of empathy’*. (P8:47:F) **Quote 2:** *‘They knew my name before they came in [to the room]… they were serious about me. I wasn't just the woman in bed 3…’* (P27:57:F) **Quote 3:** *‘… that [experience] will hold me in good spirit if I need to go back there sometime in the future’*. (P29:70:M) **Quote 4:** *‘They [Emergency Department Doctors] acknowledged that I know my body, and I've been dealing with this for a while, and I kind of know where I am and what needs to be done to treat it… And it wasn't just that one doctor, it was every doctor that I saw that acknowledged that I knew how to treat myself…’* (P4:22:F) **Quote 5:** *‘I asked if I could have a blanket, so they got one and then the lady [nurse] that I said was very kind, she actually went and heated a blanket up for me and put that over me… I think I'd marry her; she was wonderful’*. (P3:59:M) **Quote 6:** *‘If I had to rate the visit out of 10, I'd probably say a 7 or 8, because it was quite nice. The fact that the pain killers did not come at all, even though three times or four times I was asked, that probably lost big points. The coffee is probably a fraction of a point… it's the expectation of what I went there for… I did not actually go there for a meal’*. (P5:55:M)
	Being informed about and included in care	**Quote 7:** *‘They [Emergency Department staff] introduced themselves and said what they were going to do… I'm not there wondering what's going on. They told me what was going on, so that was reassuring’*. (P3:59:M) **Quote 8:** *‘It felt pretty good to be able to say I did not want one [cast]… And for them to just be honest and say,* ‘well it's not really going to do much’ *… to have the choice was nice’*. (20:23:M) **Quote 9:** *‘They [Emergency Department care providers] did not know if anything was wrong. She [physio] did say to contact my GP if it was still sore in a day or 2… it was more worry[ing] when you leave, and you do not know. You're just in pain, and thinking is it all in my head, you know?’* (P14:25:F) **Quote 10:** *‘[My] feeling of unknowing was that there did not appear to be any real inquiry as to how the pain occurred without any obvious injury, and what effect it would have and whether it could recur without warning, or whether there was any stimulus that would bring it about. I left not knowing really what I had and what I could expect as a result’*. (P7:72:M)
	Feeling confident in Emergency Department care providers	**Quote 11:** *‘…after they did all the things with my heart, my blood pressure, and then they did the urine [to check for a urinary tract infection]. And then they said there was a little infection… It made me feel safe, you think, god they look at everything, you know. And they straight away gave me an antibiotic…’* (P11:83:F) **Quote 12:** *‘…One fellow came to see me and suggested that we probably ought to have an MRI, and another fellow came along and said that maybe an MRI would not tell them much more than they already know’*. (P7:72:M) **Quote 13:** *‘He [nurse] tried to put in a cannula in my hand but it did not work, so had to go to the crook of my arm… It wasn't the best experience; he was so blasé about it… It did not feel like that was particularly something he was experienced in’*. (P1:48:F)
Being in the Emergency Department environment	Being around other patients	**Quote 14:** *‘There was an old man, kind of having a moment in the corner, which was depressing… There's a constant reminder that other people are having a worse time than me. I broke my foot, but that guy was obviously having a worse time than me’*. (P20:23:M) **Quote 15:** *‘The guy behind us was somewhat loud, probably affected by some sort of substance abuse… I thought as long as he stays nonthreatening, then everything is fine. So, I was just keeping an eye on him more so than anything else, in case things escalated’*. (P5:55:M) **Quote 16:** *‘Sometimes you wish they could just isolate you from that, because it's a bit traumatic seeing someone being escorted [out of the Emergency Department] by four hefty men, and it was a woman…’* (P16:62:F)
	Feeling comfortable	**Quote 17:** *‘…there are bathrooms there [in the waiting room] if you are waiting a while, phones – kind of those things that people might need while they are waiting’*. (P12:36:F) **Quote 18:** *‘I heard a lot of little laughter in certain spots of the room. I felt like they were just having fun… the doctors are happy, which is good to know’*. (20:23:M) **Quote 19:** *‘The other disturbing thing is when I walked in, it was like that old TV program M.A.S.H. where there were just [patients] everywhere…’* (P10:54:M)
	Having privacy	**Quote 20:** *‘… when my leg was propped on some blankets, they made sure I had a blanket over the front because I was in a dress. They made me feel really good about that, which was nice’*. (P14:25:F) **Quote 21:** *‘There was a corner [of the curtain] there that was open slightly so that the nurses and doctors could see as they walked past. It did not feel very private at that point…’* (P12:36:F) **Quote 22:** *‘My situation wasn't one that was something that had to be kept under cloak and dagger, or something that might cause embarrassment. [But] I think that if I would've been in there for some sort of very sensitive issue, I would've been whispering’*. (P5:55:M)
Variations in waiting for care	Expecting a longer wait	**Quote 23:** *‘I was very, very surprised at how quickly I was seen. Looking around the waiting room, I probably saw 10 to 12 people… You start thinking oh god I'm going to be here for six hours. And I was probably left for 10 minutes before I got called… It was quite quick’*. (P24:33:F)
	Waiting throughout the Emergency Department journey	**Quote 24:** *‘[The entire Emergency Department visit was] six‐and‐a‐half hours… It was a bit of a lengthy process. The time that I was actually seen for was probably about an hour, but the rest of the time would have been spent waiting’*. (P6:36:M) **Quote 25:** *‘After about 4 hours, they [staff] said to me that,* “we are waiting for an orthopaedic surgeon to come and look at your injury.” *And I said… I‘ve been here four hours too long now!… It was just sort of a waiting game… It's always frustrating…’* (P28:61:F) **Quote 26:** *‘One of the nurses [came and said],* “Oh, your results are back, the doctor will see you soon”*, but it still took quite a while before he came to see me. But then it's night shift as well and they have got to deal with all of that and see other patients. So it's kind of that in between of understanding, but frustration for yourself as well’*. (P18:33:F)
	Receiving timely care	**Quote 27:** *‘[I waited in the waiting room] a minute and a half, two minutes maybe… I felt so important!’* (P5:55:M) **Quote 28:** *‘Probably, the best was… the immediate attention that I received; that I did not have to wait around… Everything was one after another; everything happened’*. (P21:57:M) **Quote 29:** *‘…[care providers] got me Codeine straight away because I was crying in pain at that stage from moving from chair to bed. So it was good that they were straight onto it, knowing how sore it was. I did not really have to ask for anything’*. (P14:25:F) **Quote 30:** *‘…I had to go up and ask for it [pain relief] … I felt like I was a bit of a burden, [but] I needed it because I was getting a bit sore’*. (P6:36:M)
Having a companion in the Emergency Department	Not feeling alone	**Quote 31:** *‘They [Emergency Department staff] had no problem with my girlfriend coming in and then a couple of mates that came in and my dad came in… It definitely made time go a lot faster as well. It was good having everyone there…’* (P13:19:M) **Quote 32:** *‘It's not the nicest place to be, because no one ever really looks happy in there [Emergency Department waiting room]. But most of the time, I was just talking to my mate and just talking about what had happened’*. (P30:35:M) **Quote 33:** *‘The opportunity to have somebody else in there with me would have been good. Because it's always good to have someone else to listen to what's going on’*. (P26:38:F) **Quote 34:** *‘You've spent a whole half of the day in hospital, or in the Emergency Department. You're probably not thinking straight, so it would be handy to have another person there for that last consult, or that last discussion’*. (P25:62:F)
	Observing care providers engage with companions	**Quote 35:** *‘…The doctor came in and did an ultrasound, and my mum was excited… she mentioned that she had not seen the baby before. So, he [doctor] went and did a full thorough ultrasound and showed her [the baby's] feet… [He] went out of his way to make sure she was comfortable [and] was explaining… different things to her’*. (P4:22:F) **Quote 36:** *‘They [Emergency Department staff] acknowledged her [wife] presence, it wasn't like they ignored her or anything like that. But they did not involve her or ask her any questions… Given that it was a simple fall over and hurt yourself type of thing, they did not need to involve her as such’*. (P5:55:M) **Quote 37:** *‘…the nurse's did not acknowledge they [mum and partner] were there; they kind of pushed them out of the way when they needed to get their stuff done’*. (P4:22:F)

Abbreviations: ED, Emergency Department; GP, General Practitioner; MRI, Magnetic resonance imaging.

#### 4.2.1 Caring relationships between patients and Emergency Department care providers

Caring relationships occurring between patients and Emergency Department care providers involved both physical and emotional aspects of care. This met participants' health and well‐being needs (i.e. the need to be looked after) and their need for companionship and comradery with care providers. These relationships contributed to patients feeling valued, cared for and welcome in the Emergency Department. Three sub‐themes underpin this theme: (i) Being treated like a person and being cared for; (ii) Feeling confident in Emergency Department care providers; and (iii) Feeling included in and informed about care.

##### Being treated like a person and being cared for

Participants described being treated like a person when receiving medical care. This care was respectful and considerate of them as an individual, not a medical condition. Interactions with care providers who demonstrated *‘empathy’*, *‘concern’*, *‘compassion’* and *‘reassurance’* were crucial to making participants feel humanized and cared for, and had an enduring impact on their overall Emergency Department experience (Table 4: Quotes 1–3). However, when participants were treated like a medical condition instead of a person, they perceived this as dehumanizing. As one participant stated *‘…it was more the talking about my injury or illness. Rather than talking to me. It was almost like I was an abscess and I wasn't a person…’* (P12:36:F).

Being treated like a person was exemplified when participants were taken seriously by care providers and not dismissed about their expertise and knowledge of their own health and body. This contributed to egalitarian relationships where participants felt free to express their thoughts and opinions (Table 4: Quote 4). Conversely, some participants experienced instances where they felt forgotten about by their care providers. For example, one participant described not receiving pain relief on numerous occasions, *‘…the fact I was offered pain medication and it wasn't delivered on three or four occasions; that is the purpose of my visit and that wasn't fulfilled’*. (P5:55:M) While some participants viewed instances of being dismissed as *‘frustrating’* and *‘comical’*, others highlighted how it made them feel like a burden and as though they did not have a legitimate reason for presenting to the Emergency Department.

Another important part of being treated like a person was *‘appreciating the little things’*, such as being offered blankets, drinks and food (Table 4: Quote 5). Some participants viewed these physical comforts as kind gestures that personalized their experience, made them feel valued and went above and beyond receiving medical care. In contrast, other participants were unconcerned about these comforts, instead focusing on the (perceived) quality of care they received (Table 4: Quote 6).

##### Being informed about and included in care

This sub‐theme describes how participants' inclusion in their care was facilitated when care providers shared information with them and maintained an open dialogue. Most participants reported being well informed about several aspects of the Emergency Department continuum of care, such as potential wait times, movements in the Emergency Department, their injury/condition, planned tests/procedures and results, treatment options, upcoming consultations with other care providers, medications, their care plan (including whether they would be discharged or admitted) and discharge education. For some, being informed alleviated the anxiety, fear, confusion and isolation they felt on first entering the Emergency Department. It also meant that, despite a sense of unfamiliarity, what was happening next in the care journey did not come as a surprise (Table 4: Quote 7). For others, being informed also offered the opportunity to be actively involved in their care, such as deciding on which treatment options they preferred. Participants valued this involvement. It also strengthened their relationships with care providers because it demonstrated that care providers prioritized tailoring care to the participants’ individual needs (Table 4: Quote 8).

However, receiving limited information diminished participants' ability to be included in their care. One participant explained that being uninformed lessened her level of involvement to that of a recipient of care. *‘It's your body and they're making all these decisions for you… It's going to happen whether you like it or not’*. (P23:74:F) Others highlighted that receiving limited information about their injury/condition left them with a sense of not knowing (Table 4: Quotes 9 and 10). Thus, receiving limited information reduced participants' feelings of inclusion during their Emergency Department experience and impacted on how prepared they felt leaving the Emergency Department.

##### Feeling confident in care providers

Participants described feeling confident when care providers *‘knew what they were doing’* (P20:23:M) and provided thorough and comprehensive care. This promoted relationships founded on trust, leading participants to feel safe and relaxed in the hands of their Emergency Department care providers (Table 4: Quote 11). Yet, some described instances where their confidence in care providers faltered, especially when they received conflicting advice, leading them to feel confused (Table 4: Quote 12). Others perceived care providers were acting blasé, which made them question the extent to which they knew what they were doing (Table 4: Quote 13). Diminished confidence had a lasting impact on participants' experiences, leading them to second guess other aspects of their Emergency Department care.

#### 4.2.2 Being in the Emergency Department environment

This theme describes how the Emergency Department environment––particularly the tangible features and atmosphere of the environment––influenced participant experiences. Three sub‐themes underpin this theme: (i) Being around other patients; (ii) Feeling comfortable; and (iii) Having privacy.

##### Being around other patients

Participants actively observed the health conditions, behaviours and demeanour of other patients in the Emergency Department. When observing the health condition of other patients, some participants reflected with compassion and empathy, acknowledging that their own condition and circumstances were better than that of others (Table 4: Quote 14). One participant described seeing a young woman experiencing mental health issues as *‘probably the worst part of being in the Emergency Department’*. (P15:60:M).

Participants also reflected on the behaviour and demeanour of other patients that made them feel unsafe and as though they needed to be on guard. Situations that participants described as *‘confronting’*, *‘stressful’*, *‘distressing’* and *‘traumatic’* tended to occur in the waiting room and ambulance bay (Table 4: Quote 15). Though not all participant experiences were influenced by other patients, those whose experiences were described a heightened level of anxiety (Table 4: Quote 16).

##### Feeling comfortable

Feeling comfortable included how tangible features of the Emergency Department and the general atmosphere of the environment shaped participant experiences. Tangible features of the Emergency Department environment that contributed to participant comfort included clean waiting and treatment areas, suitable temperature control and useful facilities in the waiting room (e.g. bathrooms, TVs, phone chargers) (Table 4: Quote 17). A ‘fun’ Emergency Department atmosphere, which was noted when participants observed staff enjoying their work and working together, also contributed to positive experiences (Table 4: Quote 18). One participant described how this type of atmosphere made his experience *‘more enjoyable than it was at the time’* (P30:35:M), despite his injuries.

Yet not all participants described the Emergency Department environment as comfortable. In particular, the waiting room was consistently highlighted as uncomfortable because it was overcrowded (Table 4: Quote 19). The waiting room was also described as uncomfortable to wait in for prolonged periods because, *‘Some of the seats were quite cracked and in bad condition…’* (P1:48:F), and the televisions were not switched on, which left patients *‘just looking at each other, waiting…’* (P6:36:M) Others highlighted that the level of noise and cold temperatures in the Emergency Department made them physically uncomfortable.

##### Having privacy

Having privacy was important to participants, especially not being observed or disturbed by other patients or individuals not directly involved in their care. In many instances, participants' privacy was influenced by the behaviours of their care providers, such as whether they spoke quietly to ensure confidentially, and undertook physical examinations in a private manner. One participant described how having privacy instilled a sense of confidence about the caring nature of her care providers (Table 4: Quote 20). Conversely, another described an instance where staff failed to optimize her privacy, leading her to feel uncomfortable and as though her dignity had been compromised (Table 4: Quote 21). While the lack of privacy was disconcerting for some, other participants were unconcerned, particularly about whether others overheard them sharing information with their care providers (Table 4: Quote 22).

#### 4.2.3 Variations in waiting for care

Variations in waiting for care described how quickly (or not) care progressed. Participants described perceived delays in the initiation of their Emergency Department care (waiting in the waiting room), and in the progression of their Emergency Department care (waiting throughout their Emergency Department journey). Conversely, they also described aspects of their care that occurred quicker than they anticipated, highlighting that participant expectations influenced their experiences of waiting in the Emergency Department. Three sub‐themes underpin this theme: (i) Expecting a longer wait; (ii) Waiting throughout the Emergency Department journey; and (iii) Receiving timely care.

##### Expecting a longer wait

A long Emergency Department wait time was expected by many participants, influenced by past experiences of waiting and how busy the Emergency Department appeared to be. Many participants reflected that their current experience of waiting was better than their past experiences, noting how surprised they were (Table 4: Quote 23). One participant who went into the Emergency Department *‘prepared with some books and things… knowing that generally emergency departments have quite a wait’*, described that *‘based on how busy it was, it didn't feel like I had to wait a long time… they [Emergency Department care providers] seemed like they were quite proficient’*. (P12:33:F) Thus, when participants' expectations about long waits in the Emergency Department waiting room were disproved, their overall experience improved.

##### Waiting throughout the Emergency Department journey

Waiting was a phenomenon that occurred throughout participant Emergency Department journeys. Participants described waiting at several junctures in their Emergency Department experience, including waiting to be triaged, waiting to see care providers, waiting for tests/procedures to occur, waiting for results, waiting to be moved to different sections of the Emergency Department and waiting to be discharged or admitted. Some participants described how the experience of waiting throughout the Emergency Department journey made them feel that they were in the Emergency Department for too long. This was more pronounced when they actively engaged with their care providers for only a portion of their Emergency Department stay, as described by a participant waiting to learn the outcome of an injury (Table 4: Quote 24). In particular, some participants found waiting frustrating as they felt it was delaying their stay, preventing them from knowing what was medically wrong, and knowing whether they would be discharged home or admitted (Table 4: Quote 25).

Yet despite the frustration of some, most participants were understanding and even empathic towards the reasons they needed to wait. Participants understood that the Emergency Department was busy, and that Emergency Department care providers were under pressure, and providing care to many patients. Thus, participants recognized the need to wait for care, which they were happy to do, often describing how they regulated their feelings of frustration with understanding towards the pressures on care providers (Table 4: Quote 26).

##### Receiving timely care

In contrast to waiting throughout the Emergency Department journey, receiving timely care was described by participants as having a minimal wait in the Emergency Department waiting room, and an experience that progressed in a timely fashion. Receiving timely care made patients feel prioritized, legitimizing their reason for presenting to the Emergency Department and positively impacting their experience (Table 4: Quotes 27). One participant even described how receiving timely care was the best part of his Emergency Department experience (Table 4: Quote 28).

A major aspect of timely care related to participants' receipt of analgesia. Participants experiencing pain and discomfort described this as a memorable aspect of their Emergency Department experience, and for many, the main reason for their presentation. Thus, receiving timely analgesia improved their comfort levels, and care providers' responsiveness made participants feel they were genuinely cared for (Table 4: Quote 29). Participants who did not receive timely analgesia, however, became frustrated because it was their pain and discomfort that prompted them to initially seek emergency care. This had an overall impact on their Emergency Department experience, leaving them feeling dismissed and burdensome (Table 4: Quote 30).

#### 4.2.4 Having a companion in the Emergency Department

Participants described a companion as either a partner, friend or family member who spent a substantial amount of time with them in the Emergency Department. This played an important role in positively contributing to many participant experiences. Two sub‐themes underpin this theme: (i) Not feeling alone; and (ii) Observing staff engage with companions.

##### Not feeling alone

Not feeling alone describes how having a companion in the Emergency Department provided participants with support and company. Companions played a critical role in some participant experiences as they helped pass the time and provided someone with whom participants could communicate (Table 4: Quote 31). One participant described how having his companion in the Emergency Department waiting room alleviated the discomfort he felt being in the Emergency Department (Table 4: Quote 32).

However, some participants were unable to have a companion with them (due to COVID‐19 restrictions) and noted how this impacted their Emergency Department experience. Namely, that there was no one else to hear discharge advice, and no one to ask questions that the participant had not thought to ask (Table 4: Quotes 33 and 34). Thus, companions acted as a moderator in some Emergency Department experiences, contributing to participants' sense of confidence that they had all the information they needed before leaving the Emergency Department.

##### Observing staff engage with companions

Participants identified that care provider engagement with companions included acknowledging their presence, offering them food and drinks and involving them in the discussions taking place (Table 4: Quote 35). One participant described the level of engagement her husband received. *‘… [They asked my husband] if he wanted a sandwich, if he wanted a coffee. And you know, they asked, and they were nice… They talked to both of us, which is always a nice thing’*. (P11:83:F) Participants appreciated this engagement as they believed that it made their companion more comfortable and respected as someone of importance in the experience, despite not being there to receive care. When care providers did not acknowledge or engage with companions, some participants were neutral, believing that there was no added value in consulting with their companion when they themselves could communicate with care providers adequately (Table 4: Quote 36). Other participants however, found the lack of engagement with their companion rude and dismissive (Table 4: Quote 37).

## 5 DISCUSSION

This study explored adult patient experiences in two large Australian hospital Emergency Departments. Our findings highlight that patient experiences in the Emergency Department are multifaceted, and themes are not mutually exclusive. These findings align with those of a recent review of the international literature (Bull et al., 2021), suggesting that there is a degree of consistency in Emergency Department patient experiences the world over. Additionally, the findings depict new insights about the importance of patient‐care provider relationships, informing patients about wait times, and processes of care to make them feel included in their care. This research represents a crucial step in the conceptualization of adult Emergency Department patient experiences, which will be used to support the development of a new Emergency Department patient‐reported experience measure.

Participants in this study articulated the importance of relationships with their Emergency Department care providers. The sub‐themes *Being treated like a person and being cared for; Being informed about and included in care;* and *Feeling confident in Emergency Department care providers* align with the philosophy of person‐centred care, which promotes holistic care of the person, not just their healthcare needs (Manley et al., 2011). At the core of person‐centred care is supporting and enabling patients (people) to be partners in their care (Kennedy, 2017). While there are several challenges to enabling Emergency Department patients as partners in their care, including the severity of their condition (Elder et al., 2020; Kennedy, 2017), biomedically oriented cultures (McConnell et al., 2016) and the logistics of providing holistic care in hectic Emergency Department environments (McConnell et al., 2016; Schoenfeld, Goff, et al., 2019), care providers have a responsibility to make apparent that patients, not medical tasks, are the focus of care delivery. However, evidence suggests that translating patient preferences for greater involvement in their Emergency Department care is variable and mostly based on US research (Ijaz et al., 2018; Schoenfeld, Probst, et al., 2019). Given that partnering with health consumers is a national priority in Australia (Australian Commission on Safety and Quality in Health Care, 2017), greater examination of patient preferences for shared decision‐making in Emergency Department care, as well as an assessment of the barriers and facilitators to engaging Emergency Department patients in shared decision‐making, is required.

Despite being extensively researched (Sonis et al., 2018), our study participants reported that being informed about their wait time, particularly in the waiting room, is still an ad‐hoc process that does not align with their preferences for this information. Other research demonstrates that being uninformed about waiting can lead to patients feeling frustrated and forgotten about, negatively impacting their experiences and perceived quality of care (Bull et al., 2021; Spechbach et al., 2019). Consequently, managing patient expectations around waiting in the Emergency Department is critical, particularly as *perceived* wait times are a stronger determinant of patient experiences than *actual* wait times (Nanda et al., 2012; Sonis & White, 2020). While previous studies have shown that distractions such as televisions, art and phone chargers can reduce perceived wait times (Nanda et al., 2012; Sonis & White, 2020), implementing proactive and person‐centred strategies that keep patients informed throughout their wait may be more effective (Chu et al., 2019; Fraser et al., 2017; Sonis & White, 2020). A previous study evidenced that a bundled approach to optimizing care provider‐patient communication about wait times (inclusive of an information pamphlet and social media solution) was effective at improving communication levels and reducing patient anxiety (Taher et al., 2020). However, there have been few other interventions implemented to operationalize better wait time communication between care providers and patients. This warrants greater investigation. As being informed about wait times is an important aspect of the Emergency Department experience for patients, it is also critical that future patient‐reported experience measures capture this construct, not just the perceived duration of waiting.

Participants in this study who had a companion (i.e. friend or family) with them in the Emergency Department described the value this added to their Emergency Department experience by reducing loneliness and helping the time pass. Yet some patients noted instances where Emergency Department care providers did not acknowledge their companions, which was viewed as rude and dismissive. Demonstrating person‐ and family‐centred care is a key competency for emergency nurses and physicians globally (American Academy of Pediatrics et al., 2006; Clay & Parsh, 2016; Jones et al., 2015; Royal College of Physicians and Surgeons of Canada, 2018; The College of Emergency Nursing Australasia, 2020). Moreover, the close relationships shared between patients, family members, friends and carers may also be a critical source of information to Emergency Department care providers (Boyle, 2015), particularly where these social support networks take on an advocative role for patients (Marynowski‐Traczyk et al., 2019). However, existing research suggests that family‐centred care is poorly facilitated in some Emergency Departments (Almaze & de Beer, 2017), and that companions want to have greater communication with Emergency Department care providers and more involvement in patient care (Collom et al., 2019; Hsiao et al., 2017). Given that most research to date has focused on supporting family‐centred care in paediatric Emergency Departments (Argall et al., 2021; Brown et al., 2008; Manguy et al., 2018), facilitating Emergency Department care providers' provision of respectful, accurate and timely information to adult patient companions warrants investigation. Furthermore, patient and companion preferences for involvement in Emergency Department care should be examined as to support the provision of Emergency Department care that is person‐ and family‐centred.

### 5.1 Limitations

As with all research, we acknowledge some limitations. First, there was a period of 1 to 14 days between participant recruitment and interviewing, potentially introducing recall bias. However, this period was necessary because interviewing participants while they were still in the Emergency Department would have been both impractical and unethical as it may have interrupted their receipt of care, and would have resulted in incomplete concept elicitation (i.e. their Emergency Department journey would have been incomplete). Additionally, the period between presentation and follow‐up enabled participants to recover/ get well before being contacted. Second, selection bias may have impacted recruitment as Emergency Department care providers were critical to consenting participants. The use of a maximum variation sample frame aimed to negate this. Third, all interviews were conducted over the phone due to COVID‐19 restrictions and ethical requirements. Thus, the researcher was unable to utilize participants' visual cues such as non‐verbal body language to prompt for further questioning (Sweet, 2002). However, considering the pandemic, this was a pragmatic and ethical decision which maximized participants' safety and comfort. The participant‐researcher relationship established during face‐to‐face recruitment also ensured that participants knew that their opinions were respected as valid and valuable. Finally, mental health, homeless, correctional services/ police escorted and patients potentially infected with COVID‐19 were not recruited, suggesting that the findings may not represent the experiences of all individuals that present to Australian Emergency Departments.

## 6 CONCLUSION

The findings of this study indicate that there are four critical aspects to patient experiences in the Emergency Department. These are patient‐care provider relationships; the Emergency Department environment itself; wait times and waiting in the Emergency Department environment; and the inclusion of family and friends in the care experience. Our findings posit that patient‐care provider relationships are the most important aspect of patient Emergency Department experiences. Greater efforts to engage in shared decision‐making with Emergency Department patients will be critical to promoting person‐centredness in Emergency Department services, as will investigating strategies to enhance patient‐care provider communication about wait times. These findings contribute to the holistic conceptualization of patient experiences in the Emergency Department which is the first step in the development of a new Emergency Department patient‐reported experience measure.

## FUNDING INFORMATION

This research received no specific grant from any funding agency in the public, commercial or not‐for‐profit sectors.

## CONFLICT OF INTEREST

No conflict of interest has been declared by the author(s).

## Data Availability

The data that support the findings of this study are available from the corresponding author upon reasonable request.

## APPENDIX 1 Interview guide

**Table:**

Number	Primary questions	Sub‐questions
1	From when you got there, to when you left, what happened to you while you were in the Emergency Department?	Was this the first time you presented to the ED in the past year, or had there been other occasions?
2	What was the first thing you did when you arrived?	Who did you talk to and how would you describe your experience of talking to that person? What kind of questions did they ask you? What information did you share with them? How long did the triage process take? Was there any point that you were worried about others overhearing what you were talking about at the front desk?
3	After you spoke to the front desk, what did you do next?	How would you describe the experience of waiting in the ED? What do you remember about the waiting area itself? Were you told how long you might have to wait before being seen? Were you in pain and was this managed? How long did you wait until you were called to be seen?
4	What happened after you were called to be seen?	Can you describe what you remember about the staff taking care of you? / How did they make you feel? / How did they treat you? / What was their demeanour like? What types of tests/ examinations/ procedures did you have? What types of things did the staff discuss your condition/ care/ treatment with you? Was there ever a time you felt unsure about what was happening next? Was there ever a time you felt left out of decisions about your care and treatment? How long were you in the treatment area of the ED? If your partner was there, how did the staff engage with them? What do you remember about the treatment area you were in? Did you ever feel as though your privacy was compromised? Did you have a sense of confidence in your care provider(s)? Did you get offered food or drink while you were in the ED?
5	After you received treatment, what happened next?	Were you informed about what was happening next?
6	How did you feel about going home after you visit?	Did you feel as though you were ready to leave the ED? How did the outcome of your visit impact your experience? What type of information did staff give you before you left the ED? How long did the discharge process take? How did you go getting out of the ED?
7	What was the best part of your ED experience and why?	
8	What could have improved your ED experience and why?	
9	Is there anything we might have missed throughout this conversation that you would like me to know?	

Abbreviation: ED,Emergency Department.
